# Insights Into Patients’ Perceptions of Health-Related Social Needs and the Role of the Adult Primary Care Clinic

**DOI:** 10.1177/21501319231184380

**Published:** 2023-06-29

**Authors:** Kerry K. Meltzer, Corinne M. Rhodes, Anna U. Morgan, Gillian L. Lautenbach, Judy A. Shea, Marguerite A. Balasta

**Affiliations:** 1 National Clinician Scholars Program, University of Pennsylvania, Philadelphia, PA, USA; 2 Crescenz Veterans Affairs Medical Center, Philadelphia, PA, USA; 3 Leonard Davis Institute of Health Economics, University of Pennsylvania, Philadelphia, PA, USA; 4 Division of General Internal Medicine, Department of Medicine, Perelman School of Medicine, University of Pennsylvania, Philadelphia, PA, USA

**Keywords:** social needs, perceptions, primary care, social determinants of health, cash transfer

## Abstract

**Introduction/Objectives:**

While it is well established that unmet healthrelated social needs (HRSN) adversely affect health outcomes, there has been limited evaluation in adult primary care of patients’ perceptions of how these needs impact their health and the role of the primary care provider (PCP). The objective of this study is to identify patients’ perceptions of HRSN and how PCPs could help address them. Secondary objectives include exploring the impact of goal setting and a 1-time cash transfer (CT).

**Methods:**

This qualitative study used semi-structured baseline and follow-up interviews with patients in internal medicine clinics. Adult primary care patients were included if they screened positive as having 1 of 3 HRSN: financial resource strain, transportation needs, or food insecurity. All participants completed an initial interview about their HRSN and health, and were asked to set a 6-month health goal. At enrollment, participants were randomized to receive a $500 CT or a $50 participation reward. At 6-months, patients were interviewed again to investigate progress toward meeting their health goals, [when applicable] how the CT helped, and their beliefs about the role of PCPs in addressing HRSN.

**Results:**

We completed 30 initial and 25 follow-up interviews. Participants identified their HRSN, however most did not readily connect identified needs to health. Although participants were receptive to HRSN screening, they did not feel it was their PCP’s responsibility to address these needs. Verbal goal-setting appeared to be a useful tool, and while the CTs were appreciated, patients often found them inadequate to address HRSN.

**Conclusions:**

Given the importance of identifying the social conditions that shape patients’ health, providers, and health systems have an opportunity to re-evaluate their role in helping patients address these barriers. Future studies could examine the effect of more frequent disbursement of CTs over time.

## Introduction

The Centers for Medicare & Medicaid Services (CMS) has highlighted the importance of social determinants of health (SDOH)—the range of social, environmental, and economic factors that affect a wide range of health outcomes—by mandating health care organizations conduct social needs assessments.^1^ The U.S. Department of Health and Human Services (HHS) Healthy People 2030 categories the SDOH into 5 groups: Economic Stability, Education Access and Quality, Health Care Access and Quality, Neighborhood and Built Environment, and Social and Community Context.^2^ However, it’s important to distinguish broader social conditions from individual health-related social needs (HRSN)—a term developed by CMS as part of their Accountable Health Communities (AHC) screening tool—which are the particular social and economic needs that individuals face that can affect their ability to maintain their health and wellness.^3-6^ HRSN applies to 5 core domains—housing instability, food insecurity, transportation problems, utility help needs, and interpersonal safety—and 8 supplemental domains: financial strain, employment, family and community support, education, physical activity, substance use, mental health, and disabilities.^7,8^ Importantly, it’s recommended that patients’ perspectives on their more pressing needs should be incorporated as part of a complete HRSN screening. While research has shown that both physicians and patients are accepting of screening for HRSN in the clinical setting,^9-17^ health care providers often find it difficult to address the social barriers negatively impacting their patients’ health, given that many of these disparities lie at the population-level and are upstream from clinical care.^18-20^ Referrals can be placed to community programs and government resources, which can be helpful, but may not provide a solution to individual patient challenges.

Goal-oriented care has shown to be an effective way to individualize patient preferences and needs in the primary care setting, particularly for weight loss and glycemic control in diabetes.^21-23^ Other strategies used to help patients achieve their goals include peer support groups or app-based digital health strategies, although evidence is mixed in terms of their effectiveness.^21-26^ A newer idea, with limited empirical support in adult medicine, is the idea of a 1-time cash transfer (CT) to help patients achieve their health goals in addition to addressing their HRSN. In the U.S., research evaluating the impact of CTs have mostly been in children and have shown promising results for low-income families.^27-29^

While we know there is endorsement for HRSN screening,^16-18^ there’s been limited evaluation in adult primary care of patients’ perceptions of how HRSN impact their own health.^12^ In this study, we explored participants’ perceptions of HRSN and how primary care providers (PCPs) could help address these needs. We also asked participants to set 6-month health goals, and for a subset of patients, we explored the impact of a 1-time CT.

## Methods

### Study Design

We conducted interviews exploring HRSN and health at 2 internal medicine primary care practices at a large academic medical center in Philadelphia. At the time of enrollment, all participants were interviewed about their health—including how their HRSN were impacting their health—and were also asked to identify a 6-month health goal. Participants were randomized to receive either a $500 CT at enrollment or a $50 participation reward. At 6-months, patients were interviewed again to investigate progress toward meeting their health goals, [when applicable] how the CT helped them reach their health goal and their thoughts about the role of PCPs in addressing HRSN. This study was approved by the University of Pennsylvania School of Medicine Institutional Review Board.

### Study Population

Eligible participants were 18 years and older and screened positive as having at least 1 of 3 HRSN: financial resource strain, transportation needs, or food insecurity. From this pool, patients were eligible if they had Medicare or Medicaid and lived in a Philadelphia zip code where median annual household income was less than $50 000. All participants had at least 1 chronic medical condition including obesity, diabetes, hypertension, chronic kidney disease, coronary artery disease, chronic lung disease, were an active smoker, or had a history of a stroke. Patients who were unable to provide verbal consent due to mental disability or illness were excluded.

### Subject Recruitment

Through an electronic health record (EHR), we used a convenience sampling strategy to screen eligible participants who had a scheduled visit with their PCP between November 2021 and March 2022. The research assistant approached the patient at the time of the visit or soon after and interested participants were consented. All participants were informed during the consent process that they would receive a cash reward for participating but neither group was informed of the quantity until after they had been consented and randomized.

### Data Collection

Demographic and personal health information were collected using REDCap electronic data capture tools. Detailed chart reviews of EHR documentation were performed by trained research assistants. Interviews were conducted either in-person or over a secure virtual platform. As part of the interviews, participants were asked to rate their quality of health on a scale from Excellent (1) to Poor (5).^30,31^

### Qualitative Analysis

A semi-structured interview was developed by the research team with several goals. Open-ended questions were designed to explore the lived experiences of participants reporting HRSN. Participants were asked to reflect on how that need affected their health and the role of their PCP in addressing those needs. Participants were then asked to set a personal health goal for the next 6 months. CTs were distributed after setting the health goal and completion of the initial interview. At the 6-month interview, those who received a CT were asked how it impacted their ability to address their HRSN and health. Audio-recorded interviews were conducted by 1 of 2 trained health-services research assistants and were transcribed using the HIPAA compliant transcription firm ADA Transcription. After all interviews were completed, rapid qualitative analyses and coding were independently performed by a team of 2 internal medicine physicians and a qualitative consultant. We used the constant comparative method to identify emerging themes and thematic summaries were individually documented by each of the 3 team members.

## Results

Sample: A total of 30 patients were included in the study (Table 1). The median age was 56.4 years (IQR 45.3-68.1), 70% were female, and 90% were Black. The most frequent comorbidities were hypertension (70%), type 2 diabetes (53%), and obesity (48%). On a scale of of Excellent (1) to Poor (5), participants rated their quality of health at a 3.6 (SD 1.0). Half of participants (n = 15) were randomized to receive CTs. Eighty-three percent (n = 25) of participants completed interviews at 6-months, 14 (93%) who had received CTs and 9 (60%) who did not.

**Table 1.** Baseline Characteristics.

Characteristic	Overall (n = 30)
	n (%)
Age, median (IQR), years	56.4 (45.3-68.1)
Female	21 (70.0)
Race	
White	3 (10.0)
Black	27 (90.0)
Ethnicity	
Non-Hispanic	29 (96.7)
Hispanic Latino	0
Patient declined	1 (3.3)
Comorbidities	
Hypertension	21 (70.0)
Type 2 diabetes	16 (53.3)
Obesity	14 (46.7)
Smoker	11 (39.3)
Chronic kidney disease	7 (23.3)
Coronary artery disease	5 (16.7)
Chronic lung disease	4 (13.3)
	Mean (SD)
BMI	36.7 (11.6)
Blood pressure, mmHg	128.9/78.2 (16.2/9.5)
Hemoglobin A1C, %	6.9 (2.3)
Number of PCP visits in past 6 months	2.7 (2.2)
Self-rated quality of health^a^	3.6 (1.0)

Abbreviation: SD, standard deviation.

a Participants’ were asked, “In general, would you say your health is Excellent (1), Very Good (2), Good (3), Poor (4), Very Poor (5).”

Themes: Through the baseline and follow-up interviews, we organized our results into 4 sections, consistent with interview organization: reflections on the effect of HRSN on health, the role of PCPs in addressing HRSN, whether participants achieved their health goal, and the role of CTs.

## Theme #1: Participants’ Reflections on the Effect of HRSN on Health

Lack of reliable transportation was cited as a recurring difficulty for many patients. They described how it was often challenging to get to appointments and to the grocery store, and many had to rely on others for transporation because ride-sharing services or taxis were prohibitively expensive. Some patients talked about the difficulty of being unemployed, often because of a disability, and how that caused a substantial amount of financial insecurity. We asked participants how they felt their health would improve if some of their HRSN were met—specifically transportation difficulties and other financial hardships—and some agreed that it would make a difference: *“Well, it would – trust me, it would improve because I’d be able to get to these resources that I need to get the job that I want.”* Meeting HRSN, that is, being able to pay bills, also had other benefits: *“When bills are paid on time and in full it gives a overall sense of well-being because you don’t have due dates gnawing at the back of your head. . .It’s an overall good feeling to be able to pay for a bill when it comes in, instead of having to put it off until the money is available.”*

While most participants were able to describe how their HRSN were negatively impacting their life, when asked how they thought their health would specifically improve if their HRSN were met, many felt that it wouldn’t change, often because of the number or type of medical issues: *“I don’t believe my health would improve because I have so many medical issues, and it wouldn’t have anything to do with my social needs.”*

## Theme #2: Role of PCPs in Addressing HRSN

When asked how PCPs could help patients with their HRSN, many participants appreciated their physicians’ help with prescriptions, referrals, and some specifically mentioned getting connected with resources: *“I honestly don’t know what [PCPs] can do except for maybe offering resources to the community, but then again, a lot of times I feel like that people have to want to help themselves first. So I feel like a lot of times, people have to want to do better, and they have to help themselves in order to get other help.”*

However, the majority of participants did not think it was the PCP’s role to address these HRSN, and instead consistently talked about how it was their own responsibility: *“Nothing. That’s not [PCPs] job. That’s my job. Their job, along with me, is to work on whatever health deficits I have. I don’t confuse the two, but I do know that when my health is right, that I feel better and I accomplish more.”*

## Theme #3: Achieving Health Goals

Patients were asked to provide a health goal that they hoped to achieve over the 6-month time period between their initial and follow-up interviews. Many patients made goals to lose weight, exercise, eat healthier, and to be in less pain. We were able to complete follow-up interviews with 25 patients, 23 of whom had set discrete health goals. Among these 23 patients, 57% achieved their health goal. Among the 14 participants who received CTs and completed follow-up interviews, 9 of them achieved their stated health goal (64%). Among the 9 participants who did not receive CTs and completed follow-up interviews, 4 achieved their health goal (44%).

One patient achieved his goal of getting a prosthetic, which allowed him to be more mobile. He described how he previously needed to rely on friends to get to physical therapy but that after getting his prosthetic, he started using public transportation and felt less dependent on others. Another patient made a goal to lose weight and said that once she was able to start exercising more, she lost almost 20 pounds. For this patient in particular, it was the ability to get a new car—which occurred only after her old car was hit and she received money from her insurance company—that she was able to start getting to her appointments. She called the accident a “*blessing in disguise. . . Because of my back problem, I couldn’t really exercise at home the way I want to because it hurt. So the best thing that one of the doctors told me I should do is I could use the pool. So having that transportation, I was able to be there at the certain times for these – I had pool aerobics. . . So yes, the car allowed me to be at certain places I wouldn’t be able to be at a specific time needed.”*

## Theme #4: The Impact of CTs

For participants who received the CTs, we asked about the impact the $500 had on their life and particularly their HRSN. Some participants cited that it was very helpful, specifically for paying bills that they were behind on. One patient even said that it was because of that money that she was able to keep her heat on: *“It got me a little bit out of the hole and I caught up with a couple of my bills.”* The CT also helped with immediate needs like transportation: “*I ended up taking the last few dollars I had on [CT card] so I could go straight from dialysis.”*

For some, the CT had a positive impact in terms of mood and outlook: *“[It] lifted a little burden off me, mentally. . . . . So I would say that helped lift my spirits, and it helped ease some of the financial strain at the time.”* But in the big picture, the CT gave temporary relief: *“It helped me out a lot at the time, but it just went to bills that I had that were already overdue. So pretty much as soon as I got it, it was already spent.”*

Ultimately, many participants felt that it was not enough to make a significant change in their lives: *“I think, unless we do something on the social level to change the way people look at people who have needs, if you pay people fairly and make it possible for them to take care of their homes and find a good place to live, then I don’t think we would have all these issues with people being unhappy.”*

## Discussion

We explored the lived experiences of those reporting 1 or more HRSN and how that need is believed to impact health. We examined patients’ perceived role of the PCP in addressing HRSN and introduced a novel intervention in the form of a CT as a patient-directed tool that could potentially address those needs.

Participants were able to identify the barriers they were experiencing in their life and many felt that if those HRSN were met, their quality of life would improve considerably. However, most participants did not readily connect these HRSN to their own health. While participants were receptive to HRSN screening in the primary care setting, most did not feel it was the responsibility of their PCP to address these barriers. Verbal goal-setting appeared to be a useful tool for patients, as over half were able to achieve their stated health goal at the end of 6 months. We also found that patients were readily accepting of CTs in the primary care setting and a greater number of patients who received the CT were able to meet their goal Although a few patients found the CTs to be helpful to address specific needs, many felt that it was not sufficient to make a big enough impact.

Consistent with results from other studies,^9,11-15,17^ patients were receptive to discussing HRSN in a primary care setting. This study expands our understanding of existing literature on patient perspectives of HRSN screening, and specifically builds upon the emerging theme that patients do not feel it is the responsibility of health care providers to help them in addressing these needs.^12,32^ Health care providers agree that these disparities are often at the societal level.^18-20^ However, knowing that SDOH refers to the broader context in which people live, it’s important to recognize that it can be impacted by factors such as instutional bias, discrimination, and systemic racism, and that HRSN can in turn be a result of these SDOH.^6^ Many physician groups recommend the importance of physician involvement in these issues, whether as practicing physicians, health system leaders, educators, researchers, or advocates.^20,33^ Our study supports continued screening in the primary care setting, which can serve to strengthen the patient-provider relationship, destigmatize social services, and help shift the dialogue away from perceptions of individual failure and toward a public health approach.^34^

Our study is also consistent with previous literature regarding the importance of goal-setting in the primary care setting.^14-16,28^ In particular, the use of the SMART technique is encouraged, where participants choose goals which are specific, measurable, achievable, relevant, and time-bound.^35^ While there has been limited research in evaluating the effect of CTs in the adult primary care setting in the US, this study showed that there were a number of participants who found the CT to be helpful in achieving either a specific health goal or HRSN. However, many agreed that it was not enough to have a significant impact.

### Limitations

There were several limitations to our study. First, the study is subject to response bias as only those who agreed to participate are reflected which may bias toward patients who feel more comfortable discussing their HRSN. Second, due to our small sample size, the study was underpowered to evaluate the effectiveness of CT on goal-setting. Furthermore, a 1-time $500 CT may not have been sufficient for participants, and to further examine effectiveness, studies with multiple CTs over an extended period of time may be more successful. Third, while we explained the definition of “social needs” in the consenting process, the language may not have been intuitive so participants did not immediately connect social needs to financial strain which is discordant from previous research.^12^

## Conclusions

Our study adds to the literature of patient perceptions of HRSN screening and specifically their views that it is not the responsibility of the PCP in addressing those needs. Given the importance of identifying the social conditions that shape patients’ health, providers, and health systems have an opportunity to re-evaluate their role in helping patients address these barriers and what it means to successfully screen patients who have HRSN. A more comprehensive approach to HRSN screening could include increased investment in social support programs, patient advocacy, or partnering with community organizations to get patients the help they need. Future studies could evaluate the effect of more frequent disbursement of CTs over time, and their impact on HRSN and patients’ health goals.
